# Effects of Spider Venom Toxin PWTX-I (6-Hydroxytrypargine) on the Central Nervous System of Rats

**DOI:** 10.3390/toxins3020142

**Published:** 2011-02-22

**Authors:** Lilian M. M. Cesar-Tognoli, Simone D. Salamoni, Andrea A. Tavares, Carol F. Elias, Jaderson C. Da Costa, Jackson C. Bittencourt, Mario S. Palma

**Affiliations:** 1 Laboratory of Structural Biology and Zoochemistry, Department of Biology, CEIS, Institute of Biosciences, São Paulo State University (UNESP), Rio Claro, SP 13506-900, Brazil; Email: lmmctognoli@gmail.com; 2 Laboratory of Neurosciences, Institute of Biomedical Research and Brain Institute (InsCer), Pontifical Catholic University of Rio Grande do Sul (PUCRS), Porto Alegre, RS 90619-900, Brazil; Email: salamoni@pucrs.br (S.D.S.); almalencar@ig.com.br (A.A.T.); jcc@pucrs.br (J.C.D.C.); 3 Laboratory of Chemical Neuroanatomy, Department of Anatomy, Institute of Biomedical Sciences, University of São Paulo (USP), São Paulo, SP 05508-900, Brazil; Email: carol.elias@utsouthwestern.edu (C.F.E.); jcbitten@icb.usp.br (J.C.B.)

**Keywords:** 6-hydroxytrypargine, *fos* protein, immunohistochemistry, hypothalamus, hippocampus, epilepsy, NMDA, neurotoxicity

## Abstract

The 6-hydroxytrypargine (6-HT) is an alkaloidal toxin of the group of tetrahydro-β-carbolines (THβC) isolated from the venom of the colonial spider *Parawixia bistriata*. These alkaloids are reversible inhibitors of the monoamine-oxidase enzyme (MAO), with hallucinogenic, tremorigenic and anxiolytic properties. The toxin 6-HT was the first THβC chemically reported in the venom of spiders; however, it was not functionally well characterized up to now. The action of 6-HT was investigated by intracerebroventricular (*i.c.v*.) and intravenous (*i.v.*) applications of the toxin in adult male Wistar rats, followed by the monitoring of the expression of *fos-*protein, combined with the use of double labeling immunehistochemistry protocols for the detection of some nervous receptors and enzymes related to the metabolism of neurotransmitters in the central nervous system (CNS). We also investigated the epileptiform activity in presence of this toxin. The assays were carried out in normal hippocampal neurons and also in a model of chronic epilepsy obtained by the use of neurons incubated in free-magnesium artificial cerebro-spinal fluid (ACSF). Trypargine, a well known THβC toxin, was used as standard compound for comparative purposes. *Fos-*immunoreactive cells (*fos-*ir) were observed in hypothalamic and thalamic areas, while the double-labeling identified nervous receptors of the sub-types rGlu2/3 and NMR1, and orexinergic neurons. The 6-HT was administrated by perfusion and ejection in “brain slices” of hippocampus, inducing epileptic activity after its administration; the toxin was not able to block the epileptogenic crisis observed in the chronic model of the epilepsy, suggesting that 6-HT did not block the overactive GluRs responsible for this epileptic activity.

## 1. Introduction

Most spiders present neuroactive substances in their venom [1], which are characterized by different affinities by a series of different receptors and ionic neuronal channels [2]. During the predation, spiders inject neurotoxins able to cause paralysis of their prey due to the blocking actions at the neuromuscular junctions and/or at the central nervous system (CNS); generally the voltage-gated sodium (Nav) and voltage-gated calcium (Cav) channels constitute the most common targets of these toxins [2,3]. 

Spider venoms are complex mixtures of proteins, peptides and non-peptides low molecular mass organic (NPLMM) molecules (Mr < 1 kDa), which act mainly on the nervous system and present a wide range of pharmacological effects on synaptic transmission. The purpose of the present manuscript is not to focus the peptidic toxins from spider venoms, which probably represents the most intensively studied class of spider toxins. The NPLMM compounds more frequently reported in these venoms are free acids (such as citric and lactic), glucose, free amino acids, biogenic amines (such as diaminopropane, putrescine, cadaverine, spermine, and spermidine), and neurotransmitters (such as aspartate, glutamate, serotonin, histamine, γ-butyric acid, dopamine, and epinephrine) [4]. Several of these compounds are neurotransmitters, whereas others block ion channels at the neuronal level. Generally, low molecular mass neurotoxins offer great potential as neurochemical tools to investigate the nervous system. Additionally, they may constitute new models for drug-screening in pharmaceutical and agrochemical industries [5]. Despite the wide number of NPLMM compounds already characterized in these venoms, many others remain to be discovered. 

Some classes of NPLMM toxins have been reported in spider venoms, such as: (I) **acylpolyamines**: isolated from the venoms of orb-web-spiders; some of these compounds are neurotoxic and act as antagonists for different subtypes of ionotropic glutamate receptors, whereas others act on nicotinic acetylcholine receptors [5]; these toxins are non-competitive antagonists of glutamate receptors [6], and present neuroprotective actions as previously reported for JSTX-3, which blocks the epileptiform activity in hippocampal CA_1_ neurons *in vitro* [7,8]; (II) **bis-(agmatine)-oxamide**: isolated from the venom of the “fisher-spider”, *Plectreurys tristis* [9]; (III) **nucleoside-toxins**: mono or disulfated nucleoside compounds that are able to block kainate receptors and act on Cav1 calcium channels, such as the toxin HF-6 isolated from the venom of *Hololena curta* [10]; (IV) **tetrahydro-β-carbolines**: alkaloid compounds isolated from the venom of the social spider *Parawixia bistriata* [11] and from the web droplets of the orb-web-spider *Nephila clavipes* [12]; these compounds are reversible inhibitors of MAO-I and -II of mammals, are lethal to insects and are neurotoxic and convulsivant to rats [13]. 

The THβC are pharmacologically active, characterized by presenting hypotensive, hallucinogenic, tremorogenic and anxiolytic properties in humans [14]. These alkaloids have high affinity by different types of the receptors, such as benzodiazepinics [15,16], imidazolynics [17] and serotonergics [18]. 

The c-Fos protein is a member of a family of immediate early gene transcription factors; amongst the other members of this family Jun and Egr-1 proteins may be included, identified as proto-oncogenes. The basal expression of these genes is characteristically much reduced; however it may increase quickly in response to changes, such as metabolic stress or neuronal activation. The *fos-*protein is an inducible transcription factor, being used to indicate specific neuronal activation by different types of growth factors, neuroactive drugs, and seems to be correlated with changes in behavioral or physiological states. The immunostaining of *fos-*protein can provide a mapping of the post-synaptic stimulation in the central nervous system, with high resolution at a cellular level. It is important to highlight that the expression of *fos-*protein can be detected in the nuclei of neurons, but not in glial cells [19,20,21]. The number of *fos-*ir cells is directly related to the intensity of a specific stimulus, *i.e.*, the greater the stimulation, the greater the number of cells expressing *fos-*protein. In order to characterize the biochemical nature of some important receptors from the neurons of the brain regions responding to certain stimuli, a protocol of double labeling imunohistochemistry is generally used to identify and to co-localize specific sub-type of receptors, with the sites of *fos-*protein expression. While the immunohistochemistry protocol for *fos-*protein labels the nuclei of stimulated neurons, the staining for the specific receptors occurs in the soma, permitting the visualization of the double labeling of stimulate cells. 

Another strategy used to characterize the action of neurotoxins in rat brain is the investigation of the effects of these toxins in a model of chronic epilepsy *in vitro* by using patch-clamp electrophysiology in brain slices [7,22].

**Figure 1:**
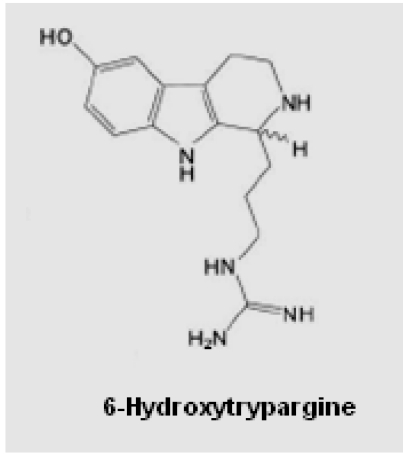
Chemical structure of the toxin 6-hydroxytrypargine (PWTX-II).

The compound 6-HT, also known as the spider toxin PWTX-I (Figure 1), is well characterized chemically [13]; however it is poorly characterized functionally. The main purpose of the present work was to investigate some actions of 6-HT in rat brain, by monitoring the expression of *fos-*protein, as a marker of the neuronal activation after *i.c.v.* and *i.v.* administration of the toxin, associated to the double labeling immunohistochemical protocol to identify the biochemical/pharmacological nature of the neurons, which are the site of action of 6-HT. The alkaloidal spider toxin was also submitted to investigation in a model of chronic epilepsy *in vitro* with hippocampal brain slices. Apparently, the results indicate that 6-HT acts in different brain regions, at the level of different sub-types of glutamate receptors and orexinergic neurons. Experiments of electrophysiology suggested that 6-HT may induce epileptogenic crisis, apparently by opening the calcium channels of glutamatergic neurons.

## 2. Materials and Methods

### 2.1. Animals

Normal 90-days old male Wistar rats, weighing 250–300 g were housed two per cage with food and water *ad libitum* in a temperature controlled (21 ± 2 °C) room on a 12 h light-dark cycle from one week prior to experimentation to allow them to acclimate to their new environment. All experiments were carried out in accordance with the guidelines of the Institutional Committee for Research and Animal Care of the University of São Paulo and the National Institutes of Health Guide for the Care and Use of Laboratory Animals [23].

### 2.2. 6-Hydroxytrypargine (6-HT) and Trypargine

The toxins 6-hydroxytrypargine and trypargine were synthesized using the protocol of synthesis of tetrahydrocarbolines, with adaptations for each compound as previously reported [13,24]. 

### 2.3. Monitoring the Expression of Fos-Protein

The method employed was performed as previously reported [25,26,27]. The guide cannula was implanted in the lateral ventricle (AP = −0.4; ML = −1.4; DV = −3.4) under anesthetic action of a cocktail (0.2 mL/100 g) containing ketamine (1 mg), xylazine (5 mg), and acepromazine (0.2 mg) seven days before the application of 6-HT. The animals were manipulated twice a day for 10 min to avoid stress on the day of the experiment. The injection cannula was introduced approximately two hours before the experiment to acclimate the animals and to minimize stress. The toxin 6-HT was solubilized in 10 µL of saline (0.9% w/v), and the compound was *i.c.v.* injected at a concentration of 10 ng/μL. The control group (n = 6) received only vehicle injection (saline: 0.9% w/v) to compare the effects of 6-HT administered in vehicle. Two hours was necessary for effective *fos-*protein induction. The animals were then anesthetized with a lethal dose of the same anesthetic cocktail used in the surgery (3 mL, intraperitoneal application) and perfused via the ascending aorta with cold 0.9% (m/v) saline (100 mL) followed by 4% (m/v) formaldehyde at pH 9.5 and 4 °C (800–1000 mL). 

The brains were removed from the skull, post-fixed during four hours in the same fixative with the addition of 20% (m/v) sucrose and then transferred to 0.02 M potassium phosphate-buffered saline (KPBS) at pH 7.4 with 20% (m/v) sucrose. The brains were sliced in four series of coronal sections (at bregma 2.70 mm, −0.30 mm, −1.80 mm, and −3.14 mm) at a thickness of 30 µm with the use of a freezing microtome and stored at −20 °C in buffered antifreeze solution [27]. 

One series of each brain slice was stained by immunohistochemistry as follows: sections were treated in 0.3% (v/v) peroxide in KPBS + 0.3% (v/v) Triton X-100 for 30 min and incubated in primary antiserum anti-*fos-*protein (PC38T IgG anti-c-Fos (Ab5) (4–17) rabbit polyclonal antibody (Calbiochem, La Jolla, CA, USA) at 1:5,000 and 3% (v/v) normal goat serum in KPBS + 0.3% (v/v) Triton X-100 for 18 h at room temperature. Sections were rinsed in KPBS and incubated for 1 h in biotinylated secondary antiserum made from goat anti-rabbit antibody (Jackson Labs 1:1000) for one additional hour in avidin-biotin complex (Vector, 1:500). Next, the sections were incubated in diaminobenzidine tetrahydrochloride (DAB; Sigma Chem Co.) and 0.01% (v/v) hydrogen peroxide dissolved in KPBS. The reaction was terminated after 2–3 min with repeated rinses in KPBS. Sections were mounted on slides and intensified with 0.005% (m/v) osmium tetroxide solution. To aid in the identification of brain regions presenting little or no *fos-*protein immunoreactive neurons (mainly in the sections of control brain slices), Nissl method of counterstaining with thionin was used [28].

Photomicrographs were acquired through a Spot RT digital camera (Diagnostics Instruments) adapted to a Leica DMR microscope and an Apple Macintosh Power PC computer using the software Adobe Photoshop 5.0. Contrast, sharpness, color balance and brightness were adjusted and images were combined in plates using Corel Draw 11 software. 

### 2.4. Double Labeling Immunohistochemistry

Dual-label immunohistochemistry was performed as reported in previous studies [25,26,27]. The sections presenting *fos-*ir neurons were rinsed in KPBS and then individually incubated with different antibodies: anti-NMR1 (1:1000; Santa Cruz Biotechnology), anti-tyrosine-hydroxylase (anti-TH) (1:1000; Santa Cruz Biotechnology) and anti-orexin (1:5000; Oncogene), overnight at room temperature. The sections were then incubated in 1,2-diaminobenzene (DAB) as chromogen and 0.01% (v/v) hydrogen peroxide in KPBS. The reaction was terminated following 2–3 min with repeated rinses in KPBS. Sections were mounted in slides and intensified with 0.005% (m/v) osmium tetroxide solution.

### 2.5. Venous Catheterization

For the intravenous (*i.v.*) administration of 6-HT, the rats were anesthetized with chloral hydrate (7%, 350 mg/kg, *i.p.*) and submitted for venous catheterisation. A Silastic catheter containing heparinized saline (10 U/mL of pyrogen-free saline, Sigma, St. Louis, MO, USA) was inserted into the femoral vein and sutured in place. The free end of the catheter was passed under the skin of the back, exteriorized between the scapulae, and plugged with a sterile wire stylet. The animals were manipulated twice a day during 10 min to avoid stress on the day of the experiment. A week later, 6-HT (100 ng kg^−1^) was intravenously applied.

### 2.6. Statistical Analysis

For the quantitative analysis of *fos-*ir and/or NMR1-ir cells, three representative slices of each brain region were chosen for each rat. All of the areas expressing c-Fos/NMR1 protein were included in the analysis. Three different animals were used in this protocol. The number of cells was counted in a defined area as follows: 0.25 mm^2^ for the piriform cortex, 0.5 mm^2^ for the lateral septal nucleus dorsal, paraventricular nucleus of the hypothalamus, dorsomedial hypothalamic nucleus, reuniens nucleus, central medial nucleus, dorsal intermediate nucleus, and 1 mm^2^ for the paraventricular thalamic nucleus and the pre-limbic cortex. The statistical analyses were performed using SigmaStat software and Student’s *t*-test was used for comparisons between groups (*p* < 0.05).

### 2.7. Electrophysiological Assay

For the electrophysiology assays normal male Wistar rats as described above were used. In order to ease pain and discomfort the animals were anesthetized via intraperitoneal (*i.p*.) injection of thiopental (40 mg/kg) before decapitation. Their brains were rapidly removed and the medial part containing the hippocampus was sectioned coronally in 400 µm thick and allowed to recover for at least 1 h before being transferred to a submersion-type recording chamber and placed on a nylon net submerged in a dissection artificial cerebrospinal fluid (ACSF) containing: 130 mM NaCl; 24 mM NaHCO_3_; 10 mM D-glucose; 1.3 mM NaH_2_PO_4_; 3.5 mM KCl; 2 mM Mg^2+^; 2 mM CaCl_2_; previously gassed with a gas mixture containing 95% O_2_ and 5% CO_2_ and pH 7.4. About two slices per rat was used in these electrophysiological recordings.

### 2.8. Electrophysiological Recordings, Data Acquisition and Analysis

Ictal-like activity and interictal discharges were induced by omitting Mg^2+^ ions from the oxygenated ACSF (0-Mg^2+^ ACSF) and pH 7.4. During this ictal-like activity the 0.1 µM 6-HT diluted in ACSF was applied by ejection through a pneumatic pump (PV830 Pneumatic Pico Pump WPI), placed next to the intracellular electrode in the CA_1_ hippocampal region. 

The recording protocols as well as stimulation for current clamp were carried out using an *in vitro* electrophysiological system [29], inside a Faraday cage. Recordings were performed in the target cell on the CA_1_ hippocampal layer, by using borosilicate micropipette (electrode impedance 80–100 MΩ) filled with 3 M potassium acetate for intracellular recordings (IC) or ACSF (5–10 MΩ) for extracellular recordings (EC) as previously reported [7,8]. Cyber Amp 380 (Axon Instruments Inc., USA) programmable signal conditioner was used, and current-clamp recordings were performed using an Axoclamp-2B amplifier (AxoClamp 2B, Axon Instruments Inc., Union City, CA, USA). The parameters used to verify the viability of a neuron were membrane potential, input resistance and action potential amplitude. Only neurons with membrane potential of at least −50 mV and action potentials with amplitude higher than 50 mV were studied. Electrophysiological variables were measured as previously described [30]. Briefly, 200 ms negative and positive current pulses were injected into the cell (−0.7 to +0.7 nA) in 0.1 nA increments. The input resistance was calculated by plotting the steady-state voltages *versus* negative current amplitudes and measuring the slope of a linear regression of the plot. 

Furthermore, only slices in which ictal-like activity was present after 30 min perfusion with 0-Mg^2+^ ACSF were studied. The data were monitored and recorded on a personal computer via the AxoScope software (Axon Instruments) and analyzed off-line with the Origin 5.0 software (Microcal Software Inc.). 

The electrophysiological protocol for the model of 0-Mg^2+^ ACSF consisted of: (A) perfusion of cells with normal ACSF for 30 min; (B) perfusion with 0-Mg^2+^ ACSF (30 min); (C) application of 6-HT (ejection); and (D) Washout of 6-HT with normal ACSF during 10 min. 

## 3. Results

### 3.1. Imunohistochemistry Assays

**Figure 2:**
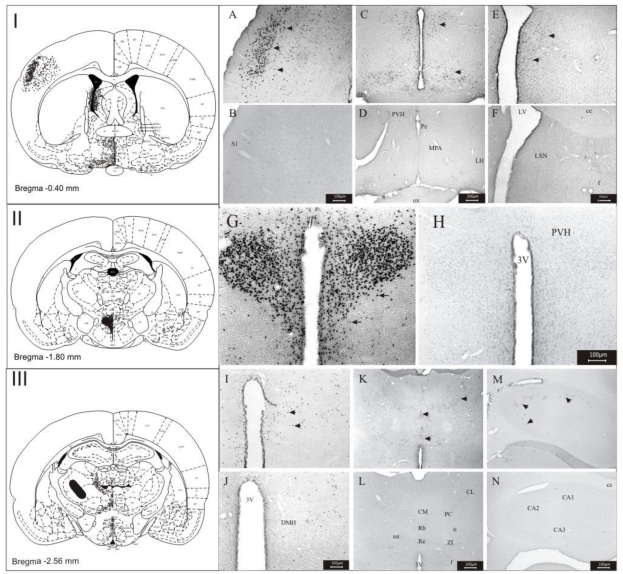
Photomicrography showing the areas of *fos-*expression after administration *i.c.v.* of the 6-HT and controls (saline). Sensorial cerebral cortex 6-HT (**A**) and control (**B**); lateral hypothalamic area and periventricular nucleus: 6-HT (**C**) and control (**D**); lateral septal nucleus: 6-HT (**E**) and control (**F**); paraventricular nucleus of the hypothalamus: 6-HT (**G**) and control (**H**); dorsomedial nucleus of the hypothalamus: 6-HT (**I**) and control (**J**); nuclei thalamus, paraventricular nucleus of the thalamus, reuniens, central medial, rhomboid, centrolateral, paracentral and zona incerta: 6-HT (**K**) and control (**L**); hippocampus: 6-HT (**M**) and control (**N**). Abbreviations: 3v: third ventricle; CA1: hippocampal region of CA1; CA2: hippocampal region of CA2; CA3: hippocampal region of CA3; cc: central comissure; CL: centrolateral; CM: central medial; DMH: dorsomedial nucleus of the hypothalamus; f: fornix; LH: lateral hypothalamic area; LSN: lateral septal nucleus; LV: lateral ventricle; Pe: periventricular nucleus; PC: paracentral; PVA: paraventricular nucleus of the thalamus; PVH: paraventricular nucleus of the hypothalamus, Rb: rhomboid; Re: reuniens; S: sensorial cerebral cortex; ZI: zona incerta. Scale bars: 200 μm in **C**, **D**; 150 μm in **E**, **F**; 100 μm in **A**, **B**, **G**–**N**. **I**, **II** and **III**: Schematic representation of the *fos-*ir area.

The *i.c.v.* application of the 6-HT in Wistar rats revealed the neuroactive character of this compound. The *fos-*ir neurons were observed mainly in hypothalamic regions and some thalamic nuclei. The labeling of *fos-*ir neurons were observed in the lateral hypothalamic area (LH), sensorial cerebral cortex (S), lateral septal nucleus (LSN), paraventricular nucleus of the hypothalamus (PVH), lateral pre-optic area (LPO), zona incerta (ZI), dorsomedial nucleus of the hypothalamus (DMH), in the periventricular nucleus (Pe), perifornical region (Pf), hippocampus and some thalamic nuclei: paraventricular nucleus of the thalamus (PVA), reuniens (Re), central medial (CM), rhomboid (Rb), centro-lateral (CL) and paracentral (PC) (Figure 2).

**Figure 3:**
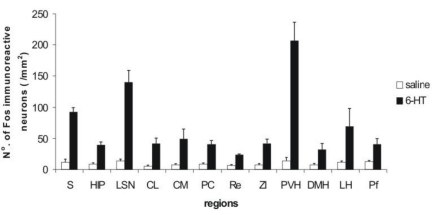
Number of *fos-*ir neurons counted in each brain area after administration of 6-HT (filled bars) or saline (open bars). Differences between both groups of animals for each encephalon region were evaluated with Students’ *t*-tests. Results are expressed as means ± S.D. (n = 6). Values of *p* < 0.05 were considered significant. CL: centrolateral; CM: central medial; DMH: dorsomedial nucleus of the hypothalamus; HIP: hippocampus; LH: lateral hypothalamic area; LSN: lateral septal nucleus; PC: paracentral; PVH: paraventricular nucleus of the hypothalamus, Re: reuniens; S: sensorial cerebral cortex; ZI: zona incerta.

Cells were counted in the twelve regions which presented the most intense labeling and compared to the control (non-treated) animals. The counting results are shown in Figure 3, which clearly permits identification of differences in *fos-*protein expression between groups of 6-HT-treated *versus* non-treated animals, indicating the action of the 6-HT on the CNS of the Wistar rats. Statistical analysis revealed that all regions presented a significant number of *fos-*ir neurons.

**Figure 4:**
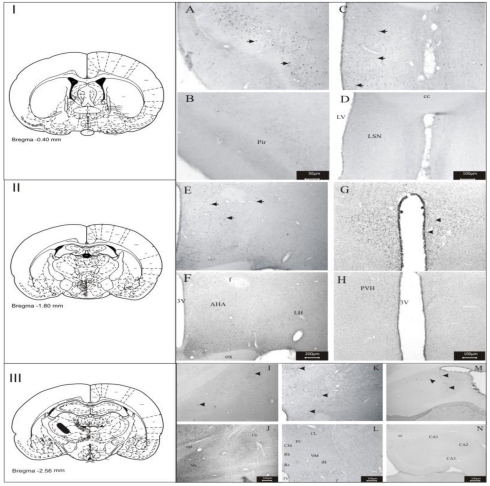
Photomicrography showing the areas of *fos-*expression after administration *i.v.* of 6-HT and controls (saline). Piriform cortex: 6-HT (**A**) and control (**B**); lateral septal nucleus: 6-HT (**C**) and control (**D**); anterior and lateral hypothalamic area: 6-HT (**E**) and control (**F**); paraventricular nucleus of the hypothalamus: 6-HT (**G**) and control (**H**); dorsomedial nucleus of the hypothalamus: control (**E**) and venom (**F**); lateral hypothalamic area and periventricular nucleus: control (**G**) and venom (**H**); central and medial nuclei of the amygdala: 6-HT (**I**) and control (**J**); paraventricular nucleus of the thalamus, reuniens, central medial, rhomboid, centrolateral and paracentral: 6-HT (**K**) and control (**L**); hippocampus: 6-HT (**M**) and control (**N**). Abbreviations: 3v: third ventricle; CA1: hippocampal region of CA1; CA2: hippocampal region of CA2; CA3: hippocampal region of CA3; cc: central comissure; Ce: central nucleus of the amygdala; CL: centrolateral; CM: central medial; DMH: dorsomedial nucleus of the hypothalamus; f: fornix; LH: lateral hypothalamic area; LSN: lateral septal nucleus; LV: lateral ventricle; Me: medial nucleus of the amygdala; Pe: periventricular nucleus; PC: paracentral; PVA: paraventricular nucleus of the thalamus; PVH: paraventricular nucleus of the hypothalamus, Rb: rhomboid; Re: reuniens; VM: ventro medial nucleus; ZI: zona incerta. Scale bars: 200 μm in **E**, **F**; 100 μm in **C**, **D**, **G**–**N**; 50 μm in **A**, **B**, **I**. **II** and **III**: schematic representation of the areas *fos-*ir.

The *i.v.* application of the 6-HT in Wistar rats showed the compound was able to cross the hematoencephalic barrier, and the labeling of *fos-*ir neurons revealed that 6-HT reached and acted in the same the regions of the brain previously observed in *i.c.v.* applications, with the exception of the anterior hypothalamic area (AHA), piriform cortex (Pir) and central and medial nuclei of the amygdala (Ce and Me)(Figure 4).

**Figure 5:**
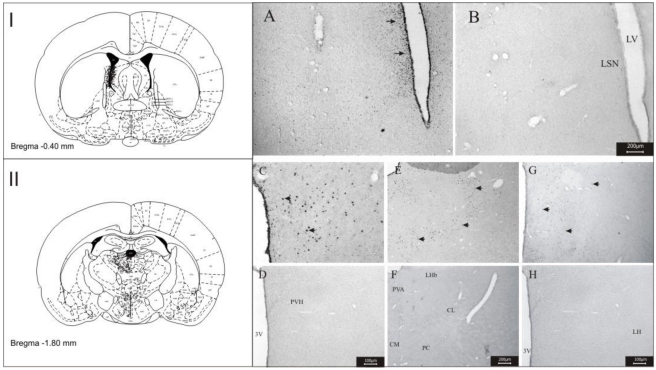
Photomicrography showing the areas of *fos-*expression after administration of the trypargine and controls (saline). Lateral septal nucleus: trypargine (**A**) and control (**B**); paraventricular nucleus of the hypothalamus: trypargine (**C**) and control (**D**); paraventricular nucleus of the thalamus, reuniens, central medial, rhomboid, centrolateral and paracentral: trypargine (**E**) and control (**F**); lateral hypothalamic area and periventricular nucleus: control (**G**) and venom (**H**). Abbreviations: 3v: third ventricle; CL: centrolateral; CM: central medial; LH: lateral hypothalamic area; LHb: lateral habenula; LSN: lateral septal nucleus; LV: lateral ventricle; PC: paracentral; PVA: paraventricular nucleus of the thalamus; PVH: paraventricular nucleus of the hypothalamus. Scale bars: 200 μm in **A**, **B**; **E**, **F**; 100 μm in **C**, **D**, **G**, **H**. **I** and **II**: schematic representation of the areas *fos-*ir.

The toxin 6-HT is a THβC as is trypargine, a neurotoxin well characterized in the literature by its action in the CNS. For comparative purposes, trypargine was used as a standard compound, and *i.c.v.* applied in the brain of male Wistar rats, as described above for the 6-HT. The results of the labeling of *fos-*ir neurons are shown Figure 5, which revealed a similar pattern of labeling induced by 6-HT. The *fos-*ir cells were observed in lateral septal nucleus (LSN), paraventricular nucleus of hypothalamus (PVH), lateral hypothalamic area (LH) and some thalamic nuclei: paraventricular nucleus of the thalamus (PVA), central medial (CM), centrolateral (CL) and paracentral (PC).

**Figure 6:**
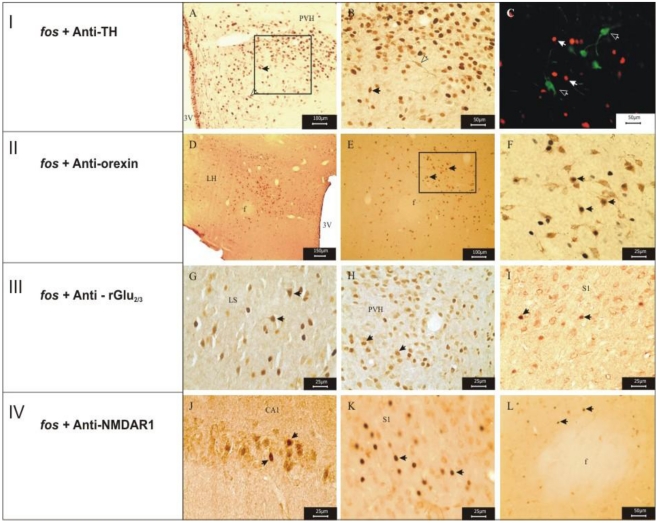
Photomicrography showing the areas of co-localization of the double labeling. fos and tyrosine hydroxylase (TH): paraventricular nucleus of the hypothalamus (**A**, **B**, and **C**); fos and orexin: lateral hypothalamic area (**D**, **E** and **F**); fos and rGlu_2/3_: lateral septal nucleus (**G**), paraventricular nucleus of the hypothalamus (**H**) and sensorial cortex (**I**); fos and NMDA-R1: hippocampal region of CA1 (**J**), sensorial cortex (**K**) and perifornical region (**L**). Abbreviations: Abbreviations: 3v: third ventricle; CA1: hippocampal region of CA1; f: fornix; LH: lateral hypothalamic area; LSN: lateral septal nucleus; LV: lateral ventricle; PVH: paraventricular nucleus of the hypothalamus, S1: sensorial cortex. Scale bars: 150 μm in **D**; 100 μm in **A**, **E**; 50 μm in **B**, **C**, **L**; 25 μm in **F**, **H**, **I**, **J**, **K**.

The identification of the biochemical/pharmacological nature of the *fos-*ir neurons to 6-HT occurred through the use of the double-labeling technique with anti-bodies anti-rGlu2/3, anti-NMR1, anti-tyrosine-hydroxylase and anti-orexin. A series of other receptors characteristics of neurons from CNS could have been used; however, the option was made for those more commonly and experimentally well characterized by this technique. The double-labeling was not observed in any *fos-*ir neuron for tyrosine-hydroxylase [Figure 6(A,B)], however, the labeling for rGlu2/3, NMR1 and orexin were observed in different regions of rat brain stimulated by 6-HT [Figure 6(C–L)], such as: CA1 hippocampal region (CA1), lateral hypothalamic area (LH), lateral septal nucleus (LSN), paraventricular nucleus of the hypothalamus (PVH), and sensorial cortex (S1). The fornix was also labeled for orexin and NMDAr1, however, this region was not *fos-*ir in 6-HT-treated animals.

### 3.2. Electrophysiological Assay

**Figure 7:**
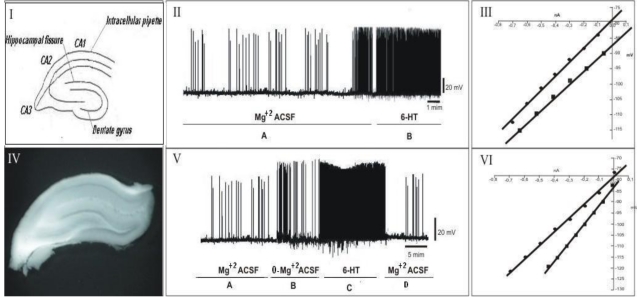
Electrophysiology assay of the 6-HT in hippocampal neurons of CA1. (**I**) Schematic slice of the hippocampus. (**II**) electrophysiologic recording of the perfusion of the 6-HT in basal state. (**III**) respective I-V curve. (**IV**) Slice of the hippocampus. (**V**) electrophysiologic recording of the perfusion in model of the 0-Mg^2+^ with the injection of the 6-HT. (**VI**) respective I-V curve.

In order to study the effect of 6-HT on the epileptiform activity induced by this toxin the intracellular potentials were recorded on hippocampal neurons from untreated rats (n = 3) kept in normal ACSF during 30 min [Figure 7(II), stage A; Figure 7(V) stages A and D] and in untreated rats kept in 0-Mg^2+^ ACSF [Figure 7(V), stage B]. A total number of six neurons were studied (two neurons per rat) Treatment with 6-HT (0.1 µM) by ejection (n = 3; 30 min) [Figure 7(II), stage B] and ejection [Figure 7(V), stage C], induced spontaneous interictal and ictal-like discharges in CA1 pyramidal neurons [Figure 6(II), stage C and Figure 7(V), stages A and B]. I *versus* V curves carried out before and following the aforementioned experimental protocol indicate that treatment with 6-HT does not significantly alter the membrane potential [Figure 7(III,VI) and Table 1]. The epileptiform activity induced by the perfusion with 0-Mg^2+^ ACSF showed in Figure 6(V), stage B is due in part, to the relief of the NMDA receptor channel from the gating effect of Mg^2+^ [8]. When 6-HT was applied in this model it was able to amplify epileptiform activity observed *in vitro* induced by 0-Mg^2+^ ACSF [Figure 6(V), stage C]. Apparently, the action is reversible since the washing with normal ACSF regenerated the pattern of spikes observed in presence of Mg^2+^ ions [Figure 7(V), stage D]. Electrophysiological neuronal properties before and after 6-HT ejection are presented in Table 1. 

**Table 1:** Effects of 6-hydroxytryptargine on electrophysiological properties of CA1 hippocampal neurons.

	Control	After 6-HT Perfusion
**Vm (mV)**	−71.63 ± 5.09	−76,07 ± 10.31
**Rin (MΩ)**	42.7 ± 7.03	54,41 ± 8,17
**Tm (ms)**	11.43 ± 2.97	10.90 ± 3.60
**Spike Amplitude (mV)**	79.33 ± 10.97	84.33 ± 12.52

Vm, resting membrane potential; Rin input resistance; Tm, Time constant of the membrane measured at Vm. All entries are expressed as means ± S.D.

## 4. Discussion

THβC compounds are endogenous in some animals, and generally are found at trace levels in mammalian brain [31]. These alkaloids probably arise endogenously in mammalian from the condensation of central nervous system indolamines (or their precursor amino acid, L-tryptophan) with an aldehyde or α-keto acid via Pictet–Spengler reaction [32]. These alkaloids act on various aspects of neurotransmission modulation, and are neurotoxic since they constitute a family of high affinity ligands of the benzodiazepine receptors, which is a sub-type of GABA receptor [33]. The THβC compounds are structurally related to the serotonin molecules [34], and because of this structural similarity these alkaloids are capable of binding to multiple receptors, such as benzodiazepinic [15,16], imidazolynic [17] and serotonergic types [35]. Trypargine, a β-carboline isolated from the skin of the African frog *Kassina senegalensis* [36], has been studied since the 1980s; it is known to cause an action-related inhibition of Na^+^ and Ca^2+^ ions current when applied in internal surface of squid axonal membranes, and also a modulatory action in 5-hydroxytryptamine-like receptors [37,38]. Alkaloidal toxins such as 1-3-guanidinopropyl-6-hydroxy-1,2,3,4-tetrahydro-β-carboline and 1-4-guanidinobutoxy-6-hydroxy-1,2,3,4-tetrahydro-β-carboline, known as PwTx-I and -II respectively, are THβC compounds isolated from the venom of the colonial spider *P. bistriata.* Meanwhile, the indolylalkaloid toxin, known as NWTx-I was isolated from the oily droplets of the web of the spider *N. clavipes*. These compounds are part of the chemical weaponry to kill/paralyze the Arthropod preys of the orb-weaving spiders [12], and are also neurotoxic, convulsive and lethal to rats [11,12,39]; however, little is known about the mechanisms of action of these toxins. 

Monoamine oxidases (MAOs) are flavin adenine dinucleotide-containing enzymes involved in the oxidative deamination of endogenous amines such as serotonin, noradrenaline and dopamine; these enzymes are ubiquitous and are localized in the outer mitochondrial membranes of mammals and arthropods [13]. Two different forms of this enzyme have been identified in mammals, MAO-A and MAO-B, which differ in their amino acid sequences, three-dimensional structures, substrate affinities and sensitivities to different inhibitors [40]. Abnormal levels of MAO-B are frequently associated with neurological disorders, while abnormalities in MAO-A activity are frequently associated with psychiatric conditions [41]. High levels of anxiety have been reported in patients bitten by the black widow spider (*Latrodectus mactans*) [42] or stung by centipedes [43]. Toxins from the venoms of other organisms, such as scorpions (*Mesobuthus gibbosus* and *Mesobuthus tamulus* *concanesis*), have been reported to inhibit the MAO enzymes from mammals [44]. Apparently, the inhibition of MAO enzymes may be responsible for the anxiety effects generally associated to these envenomations [44]. Inhibition of the deamination of endogenous amines of the victims of spider bites may be an important aspect of the envenomation mechanism of venomous arthropods; recently it was demonstrated that the 6-HT (PwTx-I) is a non-competitive inhibitor of (MAO)-A and -B [13].

Some THβC were described as psycho-stimulant compounds, being able to induce the expression of rapid transcription genes, such as the *c-*fos, in different areas of the CNS. Assays performed with *N*-methyl-β-carboline-3-carboxamide (FG-7142) revealed the expression of *fos-*protein in a series of brain areas, related to the anxyolitic behavior [45]. Meanwhile, experiments with the β-carboline harman demonstrated a thermorregulatory action in mammals [46]. The β-carboline alkaloids are capable of promoting the extracellular release of 5-hydroxytryptamine in the inner pre-optical area, a region responsible for the central regulatory mechanism of temperature [47,48]. Ibogaine, also a THβC compound, when *i.c.v.* administered in rat brains, induced the expression of *fos-*protein in the frontal-cortex, septal area (including the septal lateral nucleus), putamen-caudatum and hippocampus, suggesting a possible change in the neuroaminergic transmission, resulting from an increase of dopaminergic and/or serotonergic neurotransmission, which can be correlated to behavioral shifts [49].

The present study was undertaken in order to permit the identification of the brain regions and some of the receptors which are the target-sites of 6-HT (PwTx-I); thus, the toxin was *i.c.v.* and *i.v.* administered in male Wistar rats and the expression of *fos-*protein in different brain areas was observed: lateral hypothalamic area (LH), lateral septal nucleus (LSN), paraventricular nucleus of the hypothalamus (PVH), lateral pre-optic area (LPO), zona incerta (ZI), dorsomedial nucleus of the hypothalamus (DMH), in the periventricular nucleus (Pe), perifornical region (Pf), hippocampus and some thalamic nuclei: paraventricular nucleus of the thalamus (PVA), reuniens (Re), central medial (CM), rhomboid (Rb), centrolateral (CL) and paracentral (PC) (Figure 2, Figure 3, Figure 4, Figure 5). All these areas are directly or indirectly related to the behavior of alert and attention while the animal is exposed to the action the toxin. The importance of the *i.v.* injection of 6-HT was the possibility to compare the results obtained in this form of toxin administration, with those results got through *i.c.v*. administration; the great similarity of *fos-*ir areas observed in both experiments suggests that, that 6-HT is capable of crossing the blood-brain barrier, as previously reported for other β-carbolines [50,51,52].

Due to the structural similarity between trypargine and 6-HT, and because of previous functional characterization of trypargine [37], it was proposed to trace a comparative pattern between the labeling of *fos-*protein expression for each one of these drugs. Both compounds caused a very similar labeling pattern for *fos-*protein expression (mainly in the hypothalamic areas); thus, by comparison, it may be inferred that 6-HT may be acting preferentially at level of Na^+^ and Ca^2+^ ion channels.

The double labeling experiments were carried-out with the objective of recognizing the neuronal types expressing *fos-*protein under effect of 6-HT administration. The first double labeling reaction was performed with tyrosine hydroxylase (TH), which is an enzyme involved in the synthesis of cathecolamines in the CNS. Although, it is known that the β-carbolines are involved in the regulation of the up-taking of some biogenic amines, by inhibiting the MAO activity [13], it was observed that none of the *fos-*ir neurons belonged to the cathecholaminergic system, since no fos/TH double labeling was observed [Figure 6(I), stages A, B and C]. 

The double labeling fos/orexin revealed that about 30% of cells from the lateral and dorsal hypothalamic areas corresponded to orexinergic neurons under effect of 6-HT [Figure 6(II), stages D, E and F]. Although the orexinergic neurons are found only in the dorsal and lateral hypothalamic area, in the perifornicial nucleus, and in the dorsomedial hypothalamic nucleus, they are generally widely distributed through the projections to the hypothalamic area, cerebral cortex, pons and thalamus, amongst other CNS areas [53,54,55]. According to these authors, the distribution of the fibers suggests that the orexinergic neurons can be involved in the regulation of the feeding behavior, blood pressure, hormonal release, temperature and alert behavior.

The double labeling for the rGlu2/3 subtype AMPA-glutamate receptors (GluRs) represented a little more than 10% of the total cortical cells [Figure 6(III), stages G, H and I]. Meanwhile, the double labeling was expressive for the NMR_1_ sub-type of NMDA -GluRs) [Figure 6(IV), stages J, K and L]; 53% of the cortical cells of the S_1_ region were double labeled). These results have shown that 6-HT does not seem to be highly selective for a specific sub-type of GluR. The AMPA- and NMDA-GluRs are part of a family of ionotropic receptors; thus, the double labeling fos/NMR1 and fos/rGlu_2/3_ neurons suggests that the 6-HT seems to present a similar action on Na^+^ and Ca^2+^ channels as reported for trypargine [34]. Considering that NMDA-GluRs have an important role in the convulsive state [45], the expression of *fos-*protein in NMR_1_ neurons is possibly related to the epileptogenic properties of this β-carboline.

For the study of 6-HT in electrophysiology assays, an induced epileptiform activity model based on the removal of the Mg^2+^ ions from the ACSF was used in the hippocampal slice perfusion, determining a hyper-excitability state due to the increase of the conductance of Na^+^ and Ca^2+^ ions on NMDA-GluRs [7,8]. In addition to this model, the effect of 6-HT administration on hippocampal slices infused with normal ACSF was also investigated with the objective of studying the action of this compound on the basal state of hippocampal neurons.

The preparations of hippocampal neurons are rich in NMDA-GluRs, which consist of voltage-dependent channels partially blocked by Mg^2+^ ions [7,8]; the Mg^2+^ ions bind to the channels of NMDA-GluRs from neurons in resting potential, preventing the influx of other ions. Hippocampal slices exposed to Mg^2+^-free medium showed hyperexcitability expressed as organized seizure-like activity due to influx of Ca^2+^ ions. This hyperexcitability in experimental models of temporal lobe epilepsy is partially attributed to an increased function of a sub-population of glutamatergic synapses which uses NMDA receptors [56,57]. It is believed that not only the NMDA GluR-sub-types, but also the AMPA-GlusRs are involved in the epileptiform activity mechanisms [58,59,60], allowing changes in the permeability of Ca^2+^ ions, with important consequences for other kinetic properties of these neurons [60].

Experiments performed with 6-HT demonstrated an epileptogenic effect of this toxin on hippocampal CA1 neurons, when perfused with normal ACSF solution. In addition to this, the perfusion assays with 0-Mg^2+^ ACSF showed that 6-HT not only did not block the epileptogenic activity, but potentiated it even more, by increasing the frequency of the epileptic discharges. 

This synergic effect could be mediated by glutamatergic mechanisms. Along this line, the double labeling *fos*/NMR1 and *fos*/rGlu_2/3_ neurons suggest that the 6-HT does not seem to be highly selective for a specific sub-type of GluR and could explain the observed synergic effect since magnesium-free medium operates only in NMDA receptors. Thus, the 6-HT induced enhancement of neuronal firing without changing the membrane potential could be explained by this postsynaptic mechanism. It is most probable that a complete understanding of the actions of 6-HT on *in vitro* hippocampal neurons requires far more knowledge about cell membrane behavior than is, at present, available. This suggests that 6-HT is an epileptogenic compound (pro-convulsant) that could be acting at the level of NMDA-GluRs, preventing the blockage of Na^+^ and Ca^2+^ channels. 

The β-carbolines are also reported to cause hallucinogenic, tremorogenic and anxiolytic actions, probably due to the binding of these compounds to benzodiazepinic receptors [61,62,63,64,65,66].

The electrophysiological results obtained in the present investigation enabled the characterization of 6-HT as a probable pro-convulsant compound with reversible action once the neurons had returned to their normal initial condition after the perfusion with normal ACSF, but showed more negative membrane potentials than the initial potentials, indicating a neuronal hyperpolarization event due to the action of 6-HT [Figure 7(III,VI)]. 

The association of imunohistochemical protocols, that enabled mapping the action of 6-HT in rat brain with electrophysiological assays, contributed to the elucidation of the action of the indolyl-alkaloid toxin on the mammalian CNS. 

## 5. Conclusions

The toxin 6-hydroxytrypargine is an alkaloidal compound of the group of THβC isolated from the venom of the orb-weaving spiders, which is part of the chemical weaponry to kill/paralyze the Arthropod prey of these spiders, but also seems to be neurotoxic to mammals. In order to identify the sites of action in rat brain and to get a better understanding about some of the mechanisms involved in the neurotoxic action in mammalian brain, the action of 6-HT was investigated by *i.c.v*. and *i.v.* applications of the toxin in adult male Wistar rats, followed by monitoring the expression of *fos-*protein, combined with the use of double labeling immune-histochemistry protocols for the detection of some nervous receptors and enzymes related to the metabolism of neurotransmitters in CNS. Apparently, the toxin crosses the brain-blood barrier, acting on different brain regions, mainly in those areas directly or indirectly related to behavioral alertness and attention while the animal is exposed to the action of the toxin. Thus, it was observed that 6-HT stimulated the lateral hypothalamic area, lateral septal nucleus, paraventricular nucleus of the hypothalamus, lateral pre-optic area, zona incerta, dorsomedial nucleus of the hypothalamus, in the periventricular nucleus, perifornical region, hippocampus and some thalamic nuclei: paraventricular nucleus of the thalamus, reuniens, central medial, rhomboid, centrolateral and paracentral. 

The epileptiform activity in the presence of this toxin was also investigated. The assays were carried out in normal hippocampal neurons and also in a model of chronic epilepsy obtained by the use of neurons incubated in free-magnesium artificial cerebro-spinal fluid. *Fos-*immunoreactive cells were observed in hypothalamic and thalamic areas, while the double-labeling identified nervous receptors of the sub-types rGlu2/3 and NMR1, and orexinergic neurons. The 6-HT was administrated by perfusion and ejection in “brain slices” of hippocampus, inducing epileptic activity after its administration; the toxin was not able to block the epileptogenic crisis observed in the chronic model of epilepsy, indicating that 6-HT did not block the overactive GluRs responsible for this epileptic activity. Apparently, these results are indicative that 6-HT is an epileptogenic compound (pro-convulsant) that could be acting at the level of NMDA -GluRs, preventing the blockage of Na^+^ and Ca^2+^ channels. 

Apparently orb-weaving spiders may be predated by different birds and mammals. The existence of a polyfunctional toxin, that can act both on a web to kill/paralyze the prey Arthropods, or as a neurotoxin, causing convulsion and/or killing small mammals (depending on the dose), suggests an important chemical attribute that can be used to adapt the toxin both for hunting or defensive purposes.
